# Evaluation of Three Peptide Immobilization Techniques on a QCM Surface Related to Acetaldehyde Responses in the Gas Phase

**DOI:** 10.3390/s18113942

**Published:** 2018-11-14

**Authors:** Tomasz Wasilewski, Bartosz Szulczyński, Wojciech Kamysz, Jacek Gębicki, Jacek Namieśnik

**Affiliations:** 1 Department of Inorganic Chemistry, Faculty of Pharmacy, Medical University of Gdańsk, Hallera 107, 80-416 Gdansk, Poland; kamysz@gumed.edu.pl; 2 Department of Process Engineering and Chemical Technology, Chemical Faculty, Gdańsk University of Technology, Gabriela Narutowicza 11/12, 80-233 Gdansk, Poland; bartosz.szulczynski@pg.edu.pl (B.S.); jacgebic@pg.gda.pl (J.G.); 3 Department of Analytical Chemistry, Chemical Faculty, Gdańsk University of Technology, Gabriela Narutowicza 11/12, 80-233 Gdansk, Poland; jacek.namiesnik@pg.gda.pl

**Keywords:** sensors, biosensors, peptides, biomimetic sensors

## Abstract

The quartz-crystal microbalance is a sensitive and universal tool for measuring concentrations of various gases in the air. Biochemical functionalization of the QCM electrode allows a label-free detection of specific molecular interactions with high sensitivity and specificity. In addition, it enables a real-time determination of its kinetic rates and affinity constants. This makes QCM a versatile bioanalytical screening tool for various applications, with surface modifications ranging from the detection of single molecular monolayers to whole cells. Various types of biomaterials, including peptides mapping the binding sites of olfactory receptors, can be deposited as a sensitive element on the surface of the electrodes. One of key ways to ensure the sensitivity and accuracy of the sensor is provided by application of an optimal and repeatable method of immobilization. Therefore, effective sensors operation requires development of an optimal method of deposition. This paper reviews popular techniques (drop-casting, spin-coating, dip-coating) for coating peptides on piezoelectric crystals surface. Peptide (LEKKKKDC-NH_2_) derived from an aldehyde binding site in the HarmOBP7 protein was synthesized and used as a sensing material for the biosensor. The degree of deposition of the sensitive layer was monitoring by variations in the sensors frequency. The highest mass threshold for QCM measurements for peptides was approximately 16.43 µg·mm^−2^ for spin coating method. Developed sensor exhibited repeatable response to acetaldehyde. Moreover, responses to toluene was observed to evaluate sensors specificity. Calibration curves of the three sensors showed good determination coefficients (R^2^ > 0.99) for drop casting and dip coating and 0.97 for the spin-coating method. Sensors sensitivity vs. acetaldehyde were significantly higher for the dip-coating and drop-casting methods and lower for spin-coating one.

## 1. Introduction

The expanding knowledge of the mechanisms governing odour perception in the biological olfactory systems is accompanied by a significant progress in the field of odour biosensors [1]. Engineering synthetic materials that mimic the complex behavior of smell sense is currently the biggest challenge [2]. Improvement of biosensors’ basic parameters can be achieved by implementing materials which imitate biological materials, e.g., synthetic polypeptides, [3,4,5,6,7,8,9,10]. Selectivity of a sensor depends on the sensing material used. Generally, molecules implemented on sensor’s surface need to be designed and synthesized in such way as to achieve recognition site specific to particular analyte [11]. Owing to the ease of peptides synthesis, through modification the side chains of amino acids, the affinity for specific odorants can easily be increased. Polypeptides have some benefits owing to the fact that they retain in the solution stable secondary structures in the solution stabilized with hydrogen bonds. In an alpha-helical synthetic polypeptide, parallel and directional alignment of hydrogen bonds along the helical axis collectively produces strong electric dipole moments. This in turn, makes the peptide response to electric and magnetic fields suitable for biosensor construction. The synthesis of peptides is relatively cheap and can provide a predictable output [12]. In addition, site-specific functionalization for those molecules has already been reported [13,14]. Besides, the use of natural biological elements, e.g., olfactory tissues and proteins, as materials for the construction of biosensors, is associated with a low stability and complicated production [15]. The lifetime of peptide-based biosensors is also extended in comparison to that of easily degradable biological components and their construction is much simpler [8]. Moreover, deposition of peptides on sensor surface is possible without lipid bilayer [16]. Furthermore, the possibility of storage of peptides in refrigerator for a few months and stability of the immobilized sensitive layer make these materials the ideal for implementation in commercial odour biosensors. One of the main steps in the production of a peptide-based biosensor is immobilization on the transducer surface [17,18,19]. The primary goal of any immobilization procedure is to maintain a high activity of the sensor receptor surface. Through the use of short synthetic peptides and the appropriate method of their immobilization, it is possible to obtain a sufficiently high reproducibility and repeatability. The interactions between peptides with material surfaces are of major importance in many areas of biotechnology, including biosensors [20,21], regenerative medicine [22], implants [23], enzyme-based technologies [24], biodefense [25], etc. Surface Plasmon Resonance (SPR) and Quartz Crystal Microbalance (QCM) are two powerful label-free detection techniques that have been applied for many applications to characterize peptide-surface interactions owing to a high sensitivity and the capability to quantify peptide adsorption/desorption on surfaces in real time [26]. This type of sensors has gained remarkable importance in the fields of material science, environmental monitoring, electrochemistry and biosensors [27]. QCM can successfully be applied to analyze binding specificities, kinetics, affinities and conformational changes associated with a molecular recognition event. This technique is useful for detecting both liquids and gases and has been established as a versatile method. The thickness of a QCM determines the base resonant frequency. QCM sensors measure analyte concentrations by assessing resonant frequency change. The sorption of analyte molecules to a biochemical recognition film coated on top of the QCM resonator modifies its original loaded mass through oscillation, thus shifting the QCM resonant frequency [28]. QCM-based biosensors are increasingly used in various applications, including odorants analysis [29]. Owing to simple construction, low costs, ability to work on-line, short analysis time and suitability for a versatile, label-free analysis, this type of biosensors can be implemented in sensors matrices [12] or work independently [8,30,31,32]. A QCM biosensor includes an AT-cut quartz crystal wafer sandwiched between two metal electrodes. The use of an oscillating electric field induces an acoustic wave. The resonant frequency of the QCM depends on the change in mass on the surface of the crystals. As a result the change in frequency can be used to characterize the binding interaction between the peptide molecules and the gold electrodes. A relationship between frequency change and peptide deposition efficiency is expressed by the Sauerbrey equation [33]:

(1) $$\;\Delta f=\frac{2\Delta mf_{0}^{2}}{A\sqrt{\rho_{q}{µ}_{q}}}\;$$

where, $\rho_{q}$ and ${µ}_{q}$ are the density (2.648 g·cm^−3^) and shear modulus of quartz (2.947 × 10^11^∙g·cm^−1^·s^2^), respectively $f_{0}$ is the crystal fundamental frequency of the piezoelectric quartz crystal, A is the crystal piezoelectrically active geometrical area which is defined by the area of the deposited metallic film on the crystal, Δm and Δf are the mass and system frequency changes. Due to its extraordinary mass sensitivity, QCM sensors were originally used for gravimetric measurements [21]. According to the Sauerbrey equation, the frequency drop is proportional to the mass change, which is combined with the deposition of a given material [20]. For a typical QCM sensor with a 10 MHz frequency, a change in mass of 4.4 ng results in a frequency change of around 1 Hz·cm^−2^. The temperature dependence of the resonant frequency of an AT-cut crystal is essentially zero at 25 °C. Owing to the fact that it has a low temperature coefficient at room temperature. There is a decrease in the frequency of QCM due to deposition of mass on its surface [27]. For characterization of surface changes through deposition cycles, the Atomic Force Microscopy (AFM) is routinely used [34,35]. AFM is one of the popular techniques used in thin film characterization. Knowing the surface topography at nanometric resolution allows to investigate thin film surface characteristics [36]. Moreover, AFM can operate in ambient conditions and does not need any special sample preparation [37]. As suitable technique, extensively presented in this paper presents the optimization of octapeptide deposition on QCM gold electrode surface using three deposition methods: (i) drop casting; (ii) spin coating and (iii) dip coating. This study has evaluated the three coating techniques and several processing parameters to optimize peptide coatings on gold-coated quartz crystals. The purpose of the coating optimization was to obtain accurate and repeatable results when QCM is used as a tool to measure real-time interaction. To evaluate sensor’s sensitivity and selectivity to aldehydes, a series of experiments with acetaldehyde as a group representative, and an odorant with aromatic group, toluene, were performed. The results enabled selection of the most accurate deposition method, which would the facilitate construction of peptide-based odorant biosensors with a high degree of deposited biosensor receptor layer and optimum metrological parameters with a good selectivity and sensitivity.

## 2. Materials and Methods

### 2.1. Peptide Synthesis

The results of preliminary studies allowed to select peptide for the synthesis, mapping the aldehyde binding site in the HarmOBP7 protein, which is located in the antenna of Helicoverpa armigera [38] (specific conditions for the determination of the binding site conducted with molecular modelling and peptide length sequence evaluation will be presented in another study). Based on the properties of Odorant Binding Proteins (OBPs) and other soluble proteins involved as odorant carriers in chemical communication, can be considered as convenient candidates for imitating smell. Also OBPs binding sites, occurring in transmembrane regions, represented by peptides, are currently considered as detecting elements in odorant biosensors [39]. A peptide sequence selected for the synthesis and immobilization on secondary transducer was LEKKKKDC-NH_2_. The amide was synthesized by the solid-phase method using Fmoc chemistry strategy. The synthesis was carried out automatically on a microwave Liberty Blue™ Automated Microwave Peptide Synthesizer (CEM Corporation, Mathews, NC, USA), equipped with an IR temperature sensor and a gas cooling system. The elongation of the peptide chain was carried out in consecutive cycles of deprotection and coupling. After the synthesis, the peptide resin was dried under vacuum. Cleavage from the resin was accomplished in TFA using a scavengers mixture—TFA:EDT:TIS:water, 94:2:2:2 (*v*/*v*/*v*/*v*) for 90 min with stirring. The peptide was precipitated with cold diethyl ether and lyophilized. The crude peptide was analysed by HPLC in a water/acetonitrile gradient and purified on an X-Bridge Prep C18 column (Waters, Milford, MA, USA). Its purity was confirmed by HPLC (Varian, Mulgrave, VIC, Australia) and by using a single quadrupole mass spectrometer LC/MS system (Waters Acquity SQD). Pure fractions (> 98% by HPLC) were collected and lyophilized.

### 2.2. Method for Prefabrication of QCM-Based Biosensor

The QCM sensors (openQCM, Napoli, Italy) characterized by 10-MHz resonant frequency with polished gold electrodes and a surface finish of less than 1 micron were used. The quartz diameter of selected crystals was 13.7 mm, while the gold diameter 5.1 mm. The surface of QCM crystals was rinsed with acetone, methanol and deionized water, then dried with nitrogen until complete evaporation of solvent from the surface. Prior to coating, the gold substrates were cleaned by a strong oxidizer “piranha” (7:3 (*v*/*v*) H_2_SO_4_ (95%, Sigma Aldrich Co., St. Louis, MO, USA))/H_2_O_2_ (HPLC grade, Sigma Aldrich Co., St. Louis, MO, USA), and basic solution (1:1:3 (*v*/*v*/*v*) NH_4_OH (Sigma Aldrich Co., St. Louis, MO, USA)/H_2_O_2_/H_2_O) at 50 °C for 1 min, to remove organic materials. Before each measurement, the QCM sensor crystals were sonicated in an ultrasonic bath at 100 W at 42 kHz for 5 min (Branson Ultrasonic Co., St. Louis, MO, USA). The next step of the QCM preparation was rinsing with deionized water and ethanol and drying with nitrogen. The last step was protection of the gold surface which contacted with holder by applying a special masking fluid. For deposition studies masking the outer edge of each crystal was done, to avoid peptide aggregations near holders arms. Thick film of the fluid was placed by stainless steel syringe and removed manually after the deposition. The outer edge of each crystal was masked so that only a central circle of 10 mm in diameter was coated. Every deposition process was carried out at ambient temperature in the dark. This expected peptide monolayer, fixed to the gold surface by cysteine residues, ensured electrostatic interactions between the amino acids and odorants.

### 2.3. Peptide Deposition Methods on QCM Crystals

The purified peptide was deposited on quartz crystal microbalances using a self-assembled monolayer method (SAM). This was done through a C-terminal cysteine thiol group and resulted in SAM process on the golden electrode of the QCM crystal [40]. Basically, when a gold-coated glass plate is immersed in a thiol derivative solution, thiols begin to bind to the gold-coated glass plate to form Au-S bonds. As soon as the quantity of the Au-S bond increased, intermolecular interactions between each SAM help to form more high-density and highly oriented SAMs. Peptide solution was prepared in water/acetonitrile (1:1, *v*/*v*) as the most suitable solvent. Pure water tends to be a poor solvent for deposition due to the low vapor pressure and large surface tension. Again, some researchers suggest dimethyl sulfoxide (DMSO) as the solvent [12]. However, to avoid peptide oxidation this solvent was excluded [27]. The solvent solution was degassed with helium for 30 min before use to avoid oxidation of the thiol groups (−SH) to disulfide bonds (−S−S−). Every deposition process was carried out at ambient temperature in the dark. This expected peptide monolayer, fixed at the gold surface by the cysteine residues, promoted electrostatic interactions between the amino acids and odorants.

#### 2.3.1. Drop-Casting Method

A small volume (around 10 μL) of selected peptide solutions was deposited on the gold electrode on each side of the quartz crystal. Immobilization was performed in several replicates until no significant difference in the frequency shift was observed. Consecutive depositions were performed with an 8 h-delay. To obtain high deposition efficiency, deposition cycles were repeated until no significant difference in frequency was noticed. After each deposition, the surface of the QCM crystal was rinsed with the solvent and the biosensor was dried. Further, the chamber for drop casting was filled with nitrogen to prevent peptide oxidation and intermolecular disulphide bond formation. After each immobilization cycle, QCM sensor was rinsed by water/acetonitrile to remove uncoated peptides from the surface. The change in frequency, caused by each deposition, was measured after every cycle. Drop casting parameters were presented in Table 1.

#### 2.3.2. Spin Coating Method

In this process, a small drop of the coating material was loaded onto the center of a substrate, which was then spun at a controlled high speed. In this process, the substrate spins around an axis perpendicular to the coating area. 10 μL of the peptide solution was dropped on top of the spinning sensor. As a result, the coating material spreads towards, and eventually off, the edge of the substrate leaving a thin film of coating on the surface. Repeating the process is feasible to control the thickness of the film. The coating was done by using a developed coating machine (Gdańsk University of Technology, Gdańsk, Poland). The peptide solution was applied until the sensor frequency change has ceased. Between deposition cycles, the sensor was rinsed with water/acetonitrile and dried with nitrogen. Spin coating parameters are set out in Table 1.

#### 2.3.3. Dip Coating Method

Dip coating belongs to chemical processing techniques which overcome surpasses processings, such as physical vapor deposition and sputtering method, because of the productivity of the films and the costs of manufacturing equipment. Besides, the main advantage is a simple control of the composition and characteristics of the films and immersion parameters [41]. The dipping process was performed using commercial dipping machine (LP100dip, Kamush, Gdańsk, Poland). Peptide volume was estimated to fully cover QCM sensor during synthesis (around 7 mL). The entire dip-coating process was completed within 4 h. Between dipping cycles, the sensor was automatically dried with nitrogen and rinsed. Dip coating parameters are presented in Table 1. The QCM biosensors were stored in a vacuum desiccator after tests, until microscopic characterization.

### 2.4. Measurement Setup and Gas Chamber

**Figure 1:**
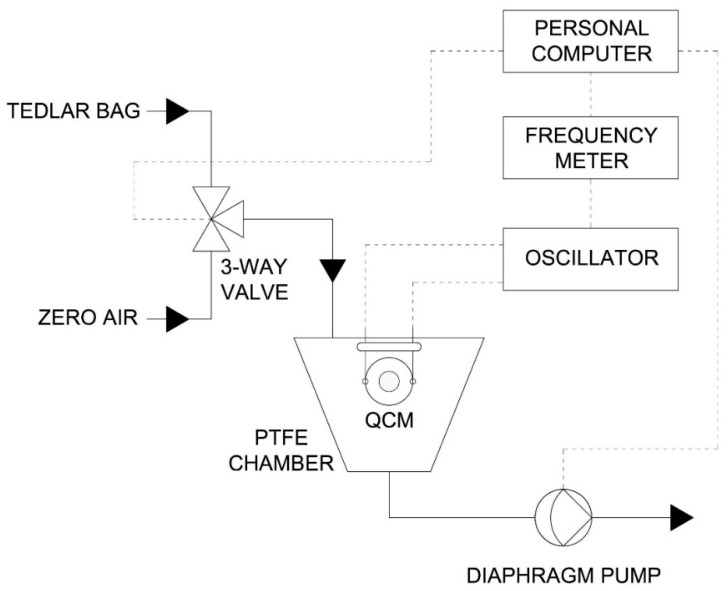
Scheme of experimental setup of standard gas solutions.

The change in frequency, caused by deposition of the peptide and also for different concentrations of aldehyde and toluene was measured. These two compounds were selected for the research owing to their affiliation to two different groups of odorous chemical compounds, aldehydes and aromatic hydrocarbons. The biosensor was connected to a standard quartz oscillator and frequency meter. Different concentrations of standard solutions were prepared in Tedlar^®^ bags via a gas mixture generator. The toluene and acetaldehyde gas mixtures were prepared in 3000 cm^3^ bags and temperature of the heated GC injector was set out at 150 °C. For the biosensor tests, five bags were filled with each calibration gas at a concentration from 1 to 10 ppm. Air volume was controlled using a mass flow controller (red-y smart series, Vögtlin Instruments, Aesh, Switzerland). A scheme of the gas mixture generator is shown in Figure 1.

**Figure 2:**
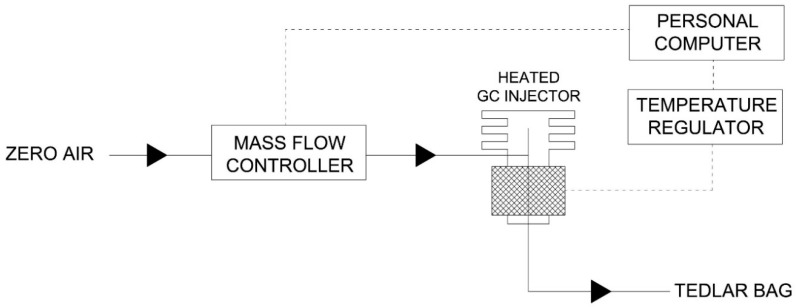
Scheme of the QCM measurement system.

The correctness of preparation of the gas standards was checked using gas chromatography coupled with flame ionization detector (430-GC, Bruker^®^, Bremen, Germany). For testing the probe response to the target gas compounds, the coated peptide-QCM biosensors were inserted into a holder and closed in a 65 cm^3^ PTFE chamber (Figure 2). The volatile fractions of the gas solutions were delivered to biosensor by a low pressure pump system. The carrier gas was pure air. After absorption of odorants on piezoelectric sensor, the chamber was purged with the carrier gas in order to remove all the adsorbed molecules.

The biosensor response was expressed as the frequency shift per mass (Hz/µg). The resonant frequency was measured with an accuracy of ± 1 Hz. Signals obtained from the system were saved on the computer and processed by QCMmeter software (Gdańsk University of Technology). The QCM was exposed to air after absorption of each analyte. The backshift of the crystal frequency to its initial value was taken as an indication of complete desorption.

## 3. Results and Discussion

### 3.1. Evaluation of Deposition

**Figure 3:**
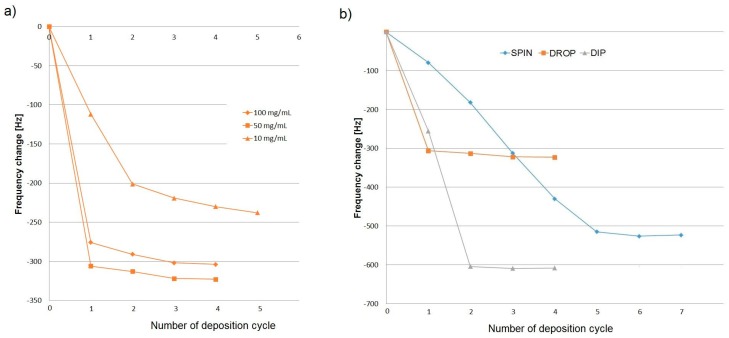
Comparison of frequency shifts during deposition cycles: (**a**) drop casting with different concentrations of peptide solution; (**b**) drop casting, dip coating and spin coating.

A quantitative evaluation of mass deposition was obtained from the difference of the quartz frequency resonance, by measuring the frequencies before and after deposition. Average depositions were calculated for three sensors of each deposition type. The deposition process was repeated until no frequency changes were noticed. In Figure 3, shifts in frequency during deposition cycles are presented.

Because the fastest saturation occurred at 50 mg·mL^−1^, this concentration was chosen for peptide suspension by further three coating methods: drop, dip and spin coating. The lowest number of cycles were required for saturation with drop casting. However, the technique ensured the lowest deposition for QCM gold electrode. At a peptide concentration of 50 mg·mL^−1^, average drop coating peptide deposition was equal to 11.36 ± 0.76 µg·mm^−2^ per side.

**Figure 4:**
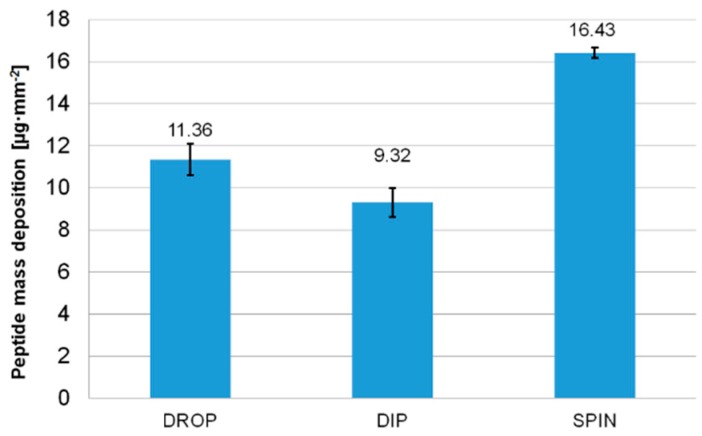
Comparison of three deposition methods in terms of the maximum peptide mass deposition.

At the lower and higher peptide concentrations (10, 50 and 100 mg·mL^−1^), the masses deposited on the crystal were respectively 8.1 ± 1.4, 11.36 ± 0.76, 10.11 ± 0.69 µg·mm^−2^. Therefore, if drop casting was chosen as a coating technique based on the ease of use, the peptide suspension should be prepared at a concentration around 50 mg·mL^−1^. Also, this concentration was used for peptide molecules deposition using other techniques (dip coating, spin coating). Peptide mass deposition on the crystal using the three methods at concentration 50 mg mL^−1^ is presented in Figure 4.

Application of multiple coatings, when comparing an increase in the suspension concentration, was the best method to increase the mass loading on the crystal. The highest mass threshold for QCM measurements for peptides was approximately 16.43 µg·mm^−2^. A comparison of the deposition techniques used for peptide molecules with Cys-terminated end is shown in Table 2.

### 3.2. Peptide-Based Sensor Film Characterization

**Figure 5:**
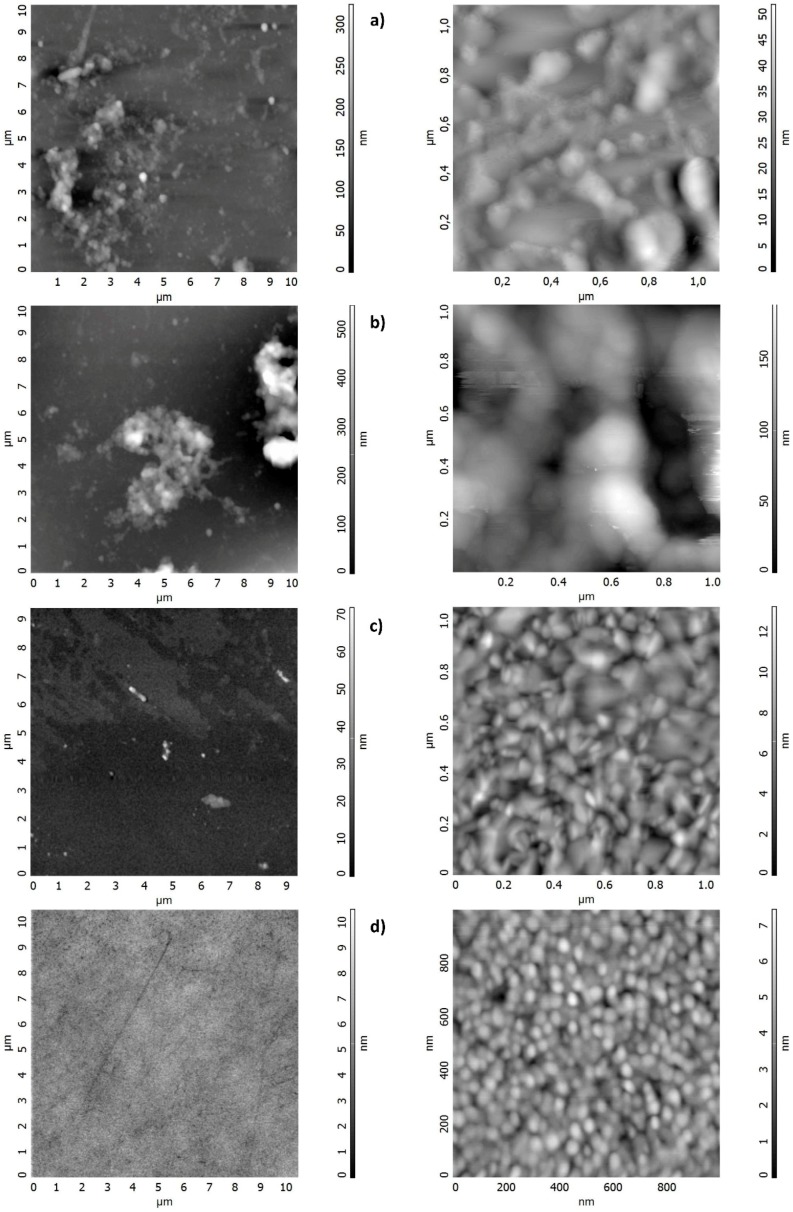
AFM images of sensors fabricated: (**a**) drop-casting (Peak-to-peak, Sy: 790.501 nm; Ten point height, Sz: 393.493 nm, Average Roughness, Sa: 152.859 nm); (**b**) dip-coating (Sy: 1128.94 nm, Sz: 557.869 nm, Sa: 161.4 nm); (**c**) spin-coating (Sy: 71.59 nm, Sz: 34.47 nm, Sa 1.97 nm); and (**d**) reference electrode: (Sy: 4.94 nm, Sz: 2.56 nm, Sa: 0.33 nm).

The AFM images were acquired to determine the surface morphology of the film on the QCM sensor. For the purpose of measurements, an AFM Ntegra Prima device manufactured by NT-MDT (Moscow, Russia) was used. In the topographic measurements, the tapping mode with the set-point equal to half-value of free oscillations amplitude was applied. The measurements were carried out using conductive probes of the NSG 01 type, manufactured by NT-MDT. The geometric dimensions of the probe lever were 125 × 30 × 2 (L × W × T/mm), while other parameters were as follows: resonance frequency: 150 kHz, spring constant: 5.1 N/m, radius of tip curvature: 10 nm. In all studies on biosensors, regardless of the deposition method, a peptide monolayer was observed (10 µm × 10 µm). AFM images indicated a uniform thin film formation for spin-coating method (d), although peptide aggregates can occur over a large area, the sensing layer was non-uniform in the dip- and drop-method, however film smoothness was acceptable. Moreover, irregular clusters of local aggregations of the peptides were seen. The largest film thickness, approximately 606 nm, was found with the dip-coating method. To show the uniformity of the layers an AFM images at 10 µm × 10 µm with basic parameters and 1 µm × 1 µm with are presented in Figure 5.

### 3.3. Peptide-Based Sensors Responses to Odorants

**Figure 6:**
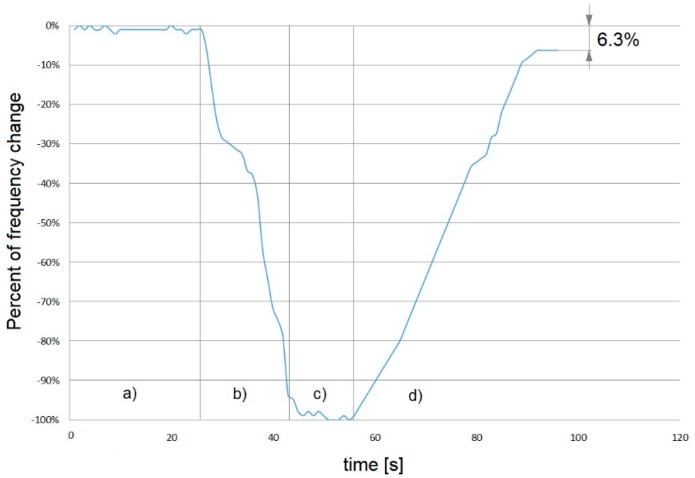
Typical response of QCM sensor (peptide drop-casting deposition) to the lowest measured concentration, 1 ppm acetaldehyde: (**a**) baseline stabilization, (**b**) gas introduction, (**c**) signal *plateau*, (**d**) return to the initial sensor state.

All sensors expressed a linear frequency decrease in the presence of increasing aldehyde and toluene concentrations (1–10 ppm) with a high (r^2^ > 0.90) coefficient of determination (Figure 6 and Table 3).

**Figure 7:**
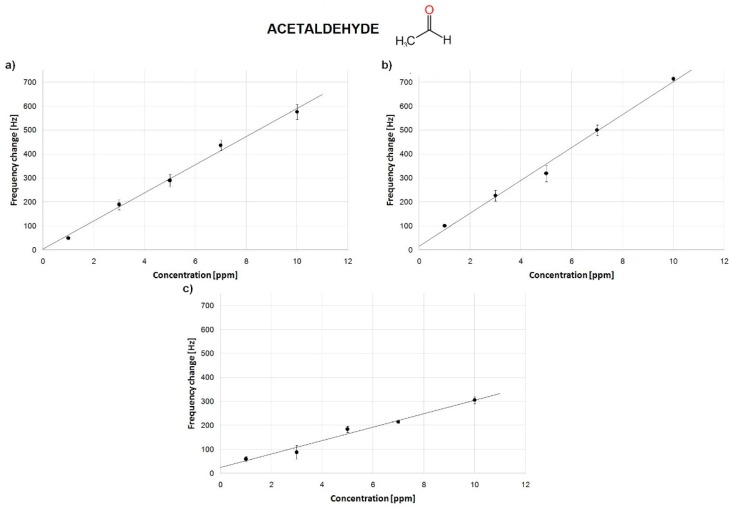
Calibration curve of sensors to acetaldehyde for different deposition methods: (**a**) dip-coating; (**b**) drop-casting; (**c**) spin-coating.

An exemplary peptide-based QCM sensor response to acetaldehyde at room temperature is presented in Figure 6. The QCM sensor’s resonant frequency decreased after the gas introduction. The rapid absorption of aldehyde molecules on the sensor receptor layer caused a quick decrease in the sensor resonant frequency. Calibration curves of the three sensors showed a satisfying linear correlation coefficients (r > 0.90) (Figure 7).

**Figure 8:**
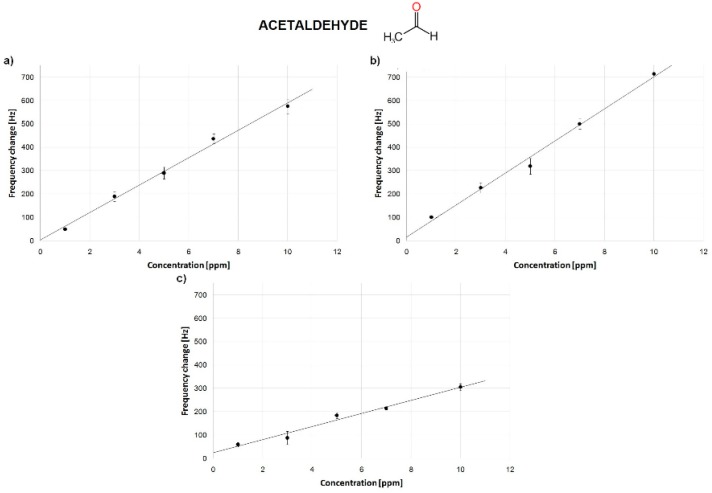
Calibration curve of sensors to toluene for different deposition methods: (**a**) dip-coating; (**b**) drop-casting; (**c**) spin-coating.

The average response was automatically calculated among three measurements. In all the measurements, the response of a bare QCM was monitored to confirm the negative response. Frequency changes between static and dynamic conditions were compared. Baseline stability during sensor stabilization was too low to reliably estimate sensor frequency response in the flow mode. Because of the improved sensor stability under static conditions, this mode was chosen. The results are presented in Figure 7 and Figure 8 and summarized in Table 3.

A similar sensitivity for acetaldehyde was obtained for the dip-coating and drop-casting-based QCM biosensors (Figure 7). On the other hand, the spin-coating-based QCM biosensors were characterized by a lesser efficiency. Calibration curves of the three sensors showed satisfying determination coefficients (R^2^ > 0.99) for drop casting and dip-coating and 0.97 for spin-coating method. Sensors sensitivity vs. toluene were significantly lower for the dip- and drop-coated methods and higher for the spin-coating one (Figure 8).

Repeatability of QCM sensors for each concentration of acetaldehyde was tested over repeated cycles at room temperature. Sensor responses were repeatable for known concentrations of aldehyde. Most of the sensor response had the coefficient of variation of less than 8%, indicating a small difference within the response cycles of the QCM sensors. The variation coefficient of 15 measurements carried out for a period of three weeks are presented in Table 4.

The limit of detection (LOD) was defined as the minimum concentration of analyte, that produces a clear peak with signal-to-noise ratio equal 3, as is a common practice in the literature [30]. Estimated LOD of the QCM sensors was about 1 ppm for acetaldehyde and about 2 ppm for toluene.

As far as the effect of humidity is concerned, the selected peptide was a component of transmembrane domain of the OBP, which has been known to be a hydrophilic region. As a reference gas, zero air from generator, with 2 ± 1% relative humidity was used. It was found that the QCM sensor sensitivity was repeatable during measurements. This suggested that the effect of humidity was insignificant, with a little to no effect on QCM sensor frequency change during measurements.

## 4. Discussion and Conclusions

Up to date, several technologies were considered for their detection by using metal oxides [42] or QCM coated with polymers [43] such as MIP [44]. As compared to various commercial aldehyde sensors’ sensitivity (~5 ppm), the peptide-based QCM sensors have shown a better sensitivity [11] and comparable to that of a sensor developed by Imashi et al. [45] and Giberti et al. [46]. A sensor with the MIP film, with a similar sensitivity for aldehyde was also developed by Debliquy et al. [44]. However, these sensors required a high operating temperature, of 250–550 °C. Balamurugan et al. [42] developed sensor with co-doped ZnO film capable to work at ambient temperature (~30 °C) with a sensitivity around 10 ppm. The estimated LODs of QCM-based sensors for acetaldehyde detection were found to be the lowest for drop casting and dip coating methods. All the methods are highly repeatable and produce uniform films over large areas. Although the lower peptide deposition yielded a uniform thin sensor film, the sensitivities and slopes of calibrations curves were much lower than those obtained with high deposition rates and a lesser uniformity. Sensors prepared using spin coating method showed higher LODs. In principle, film thickness depends on the volume of dispersion used and the particle concentration. Other examples of variables that affect the film structure are solvent substrate-wetting, properties of the solvent, evaporation rate, capillary forces associated with drying, etc. A lesser efficiency of spin-coating method in biosensor responses for acetaldehyde might be connected with those factors. One drawback of drop-casting is that even under almost ideal conditions, differences in evaporation rates across the substrate or concentration fluctuations can cause variations in film thickness. However, drop-casting is a quick and accessible method, generating thin films using relatively small volumes of coating solutions. Dip coating method seems to be the most repeatable one and allows to fabricate the most sensitive peptide-based receptor sensor layers. Taking into account SD and LOD values for acetaldehyde measurements, drop-casting and dip-coating methods are comparable with a slight predominance of dip-coating one. If we also take into consideration the fact that dip-coating can be easily automated and more homogeneous film are created, the authors suggest using this method for deposition of peptides on quartz crystal microbalance gold substrates. Based on computational data and a series of binding experiments (data not shown here) it is assumed that aldehyde binding in this peptide is realized by formation of a Schiff base through the side chains of Lys. The binding pocket is additionally stabilized by electrostatic interactions allowing to interact freely with aldehyde ligands. A series of experiments confirmed that QCM sensors with a deposited peptide can detect low acetaldehyde concentrations (low ppm), desirable for our future application. Drop-casting was the simplest of the four techniques that were tested, but the quality and reproducibility of drop cast coatings is heavily dependent on operator capability. For the future, the authors recommend to validate the developed sensor with best sensitivity on a larger dataset as well as sensor sensitivity and repeatability after longer periods (one month, six months, twelve months) need to be estimated. Humidity effect should be evaluated on real samples. QCM as an in situ tool continues to be one of the most useful and accesible methods for mass-metric control. The high sensitivity to changes in mass makes this method unique for investigation of deposition of biomaterials. The classic application of QCM as a mass-metric device for the study of propagation of different processes has been continued in recent research.

## Figures and Tables

**Table 1:** Parameters of peptide depositions techniques on QCM surface.

**Drop Casting**								
Peptide Concentration	Peptide Volume For Cycle	Number of Drop Cycles						Deposition Time
10; 50; 100 mg/mL	10 µL	3–4						8 h
**Spin Coating**								
Peptide Concentration	Peptide Volume For Cycle	Number of Spin Cycles		Spin Rate (Rpm)		Spin Acceleration (Rpm^2^)		Spin Time (S)
50 mg/mL	10 µL × 7	7		3000		100		10 × 30
**Dip Coating**								
Peptide Concentration	Peptide Volume	Number of Dip Cycles	Dwell Time (S)		Immersion Rate (Mm/Min)		Withdraw Rate (Mm/Min)	Total Time
50 mg/mL	7 mL	2–3	90		240		50	4 h

**Table 2:** Comparison of different peptide deposition techniques.

Technique	Advantage	Disadvantage
Drop casting	ease of use, quick and accessible method, easy to perform under vacuum or neutral gas atmosphere, small volume of coating solution.	reproducibility heavily dependent on operator capability, hard to obtain uniform coating and control thickness, differences in evaporation speed, concentration fluctuations.
Dip coating	good quality of the uniformity, high reproducibility, easy to control layer thickness, large area coverage, equal double side coverage, best scratch and breaks resistance.	highest waste of material, time consuming, requirement of special instrument, hard to perform under the vacuum or neutral gas atmosphere, time-consuming.
Spin coating	good uniformity, high reproducibility, good control on thickness, low cost and fast.	high waste of material, fast film drying, requirement of special instrument, hard to perform under vacuum or neutral gas atmosphere.

**Table 3:** Comparison of different deposition techniques parameters.

Deposition Method		Dip	Drop	Spin
**Mass deposition**		9.32	11.36	16.43
**Acetaldehyde**	R^2^	0.994	0.991	0.977
	Sensitivity (Hz·ppm^−1^)	58.8 ± 2.8	68.5 ± 3.9	28.1 ± 2.6
	LOD (ppm)	1.0	1.2	2.0
**Toluene**	R^2^	0.980	0.984	0.987
	Sensitivity (Hz·ppm^−1^)	6.13 ± 0.52	5.57 ± 0.49	3.10 ± 0.21
	LOD (ppm)	1.7	1.8	1.4

**Table 4:** Repeatability evaluation of the results of the prepared sensors.

Deposition Method	Dip			Drop			Spin		
**Acetaldehyde concentration (ppm)**	1	5	10	1	5	10	1	5	10
**Frequency change mean value (Hz)**	49.3	287.8	579.6	96.4	316.3	712.7	60.3	181.4	298.7
**Standard deviation (Hz)**	2.1	7.1	6.9	6.4	11.7	18.1	4.8	12.5	14.1
**RSD (-)**	4.2%	2.5%	1.2%	6.6%	3.7%	2.5%	8.0%	6.9%	4.7%
